# A Simple Model to Estimate the Number of Metal Engineered Nanoparticles in Samples Using Inductively Coupled Plasma Optical Emission Spectrometry

**DOI:** 10.3390/molecules27185810

**Published:** 2022-09-08

**Authors:** Nokwanda Hendricks, Olatunde Olatunji, Bhekumuzi Gumbi

**Affiliations:** School of Chemistry and Physics, University of KwaZulu-Natal, Private Bag X54001, Durban 4001, South Africa

**Keywords:** number of nanoparticles, gold nanoparticles, molar concentration, ICP-OES, centrifugation, phase transfer, nanomaterials, metal engineered nanoparticles

## Abstract

Accurate determination of the size and the number of nanoparticles plays an important role in many different environmental studies of nanomaterials, such as fate, toxicity, and occurrence in general. This work presents an accurate model that estimates the number of nanoparticles from the mass and molar concentration of gold nanoparticles (AuNPs) in water. Citrate-capped AuNPs were synthesized and characterized using transmission electron microscopy (TEM) and ultraviolet–visible spectroscopy (UV-vis). A mimic of environmental matrices was achieved by spiking sediments with AuNPs, extracted with leachate, and separated from the bulk matrix using centrifuge and phase transfer separation techniques. The quantification of AuNPs’ molar concentration on the extracted residues was achieved by inductively coupled plasma optical emission spectroscopy (ICP-OES). The molar concentrations, an average diameter of 27 nm, and the colloidal suspension volumes of AuNPs enable the calculation of the number of nanoparticles in separated residues. The plot of the number of AuNPs against the mass of AuNPs yielded a simple linear model that was used to estimate the number of nanoparticles in the sample using ICP-OES. According to the authors’ knowledge, this is the first adaptation of the gravimetric method to ICP-OES for estimating the number of nanoparticles after separation with phase transfer.

## 1. Introduction

In recent decades, exponential growth in metal engineered nanomaterial (ENMs) research and development has resulted in mass-scale industrial production and extensive commercialization of products containing metal ENMs [1]. The increase in utilization has led to the release of metal ENMs into the environment [2,3] Many studies have investigated the possible adverse effect of metal ENMs on human and animal health; however, an investigation into their environmental occurrence, fate, and behavior in the environment is required to strengthen risk assessment and to provide recommendations to policymakers [4,5,6,7,8,9]. There is a lack of these data on metal ENMs in a natural environment, and their environmental impact is poorly understood due to unavailability of extraction methods and well-developed robust analytical methods to analyze ENMs [10,11,12]

While the concentration of metal engineered nanoparticles (ENPs) has been reported in other forms (e.g., mass), accurate determination of molar concentration and, subsequently, the number of nanoparticles might be the most important parameters to study their fate, behavior, toxicity, and environmental impact, which have been a problem mostly for ENPs [13]. Towards solving these problems, different analytical models are being developed; most of these models are restricted to certain nanoparticles due to their dependence on the composition and sizes of nanoparticles. Different models have been developed based on these methods: gravimetric measurements, light absorption, turbidimetry, dynamic light scattering, laser-induced breakdown detection, single particle counting, induced plasma coupled-spectrometry, and optical sensing [13,14]

Reipa et al. [15] developed a micro-gravimetric method to measure the nanoparticle concentration of silver and silicon using a nanogram resolution mass sensing technique. In addition, the nanoparticles were characterized by UV-vis and TEM to obtain parameters needed in the method for molar concentration calculations. Also, Paramelle et al. [16] developed a model based on extinction coefficient data for silver nanoparticles (AgNPs) the data was obtained using UV-vis and light spectra. Their model allowed the quick estimation of the molar concentration and the sizes from the optical spectra. In another study, Levin et al. [17] proposed a method to measure the size of nanoparticles using the dynamic light scattering (DLS) analyzer to determine the number density in suspension. They concluded that the method proposed can be used to measure various nanoparticles, including non-spherical-shaped particles. However, the most used method for the analysis of metal ENPs in the environment is a single particle counting technique based on single particle inductively coupled mass spectrometry (SP-ICP-MS), which uses transport efficiency to estimate nanoparticle concentration in the sample [18,19,20,21,22,23,24,25,26,27] 

Since the rate at which metal ENPs are developed and used is rising rapidly, it is necessary to develop universal models that are simple to apply and specific to metal ENPs’ sizes and shapes. To address the need for a simple method to measure the total number of nanoparticles in the sample, a bottom-up method has been developed that starts with synthesis, characterization, spiking, extraction, separation, and quantification of nanoparticles. The data obtained are presented as a set of convenient calibration curve plots, tables, histograms, polynomial plots, and formulas. Lastly, the gravimetric method was adapted to show that the mass of AuNPs obtained from molar concentration is related to the number of AuNPs. This relationship model is expected to apply to different nanoparticle types that can be separated and analyzed by ICP-OES.

## 2. Results

Metal ENPs were synthesized using the wet chemical method. The wet chemical method is a bottom-up approach. The general approach involves the reduction of the chloroauric anion (AuCl_4_^−^) as the gold salt and silver nitrate by various reducing agents: tri-sodium citrate, ascorbic acid, and sodium borohydride. AuCl_4_^−^ during reduction by the reducing agents produces neutral metal atoms, which undergo nucleation and growth processes to form AuNPs in the presence of the capping molecule, tri-sodium citrate [28,29,30,31]. The characterisation of AuNPs was performed to confirm the sizes, shapes, and surface properties of nanoparticles.

### 2.1. Characterization of Engineered Nanoparticles

**Figure 1:**
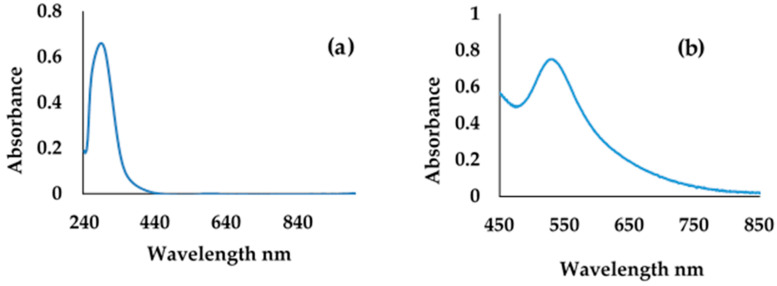
UV-vis absorption spectra of (**a**) gold salt and (**b**) gold nanoparticles.

Characterization of engineered nanoparticles with UV-vis spectrometry. The optical properties of metal ENPs were studied with UV-vis spectrometry. The spectral range of metal ENPs is encompassed within the range of the human eye, which made it easy to follow the synthesis of metal nanoparticles without extensive instrument dependence. The transmission of light through the sample was recorded as the function of the wavelength. Gold (chloroauric) salt was dissolved in Millipore water and analyzed with UV-vis. The UV-vis spectrum of gold salt is presented in Figure 1a, and it has an absorption band of 440 nm. Upon reduction of Au salt using citrate, the UV-vis scan of the reduced and stabilized AuNPs showed absorption and a shift in the absorption wavelength of absorption. The band was shifted to the longer wavelength, which confirmed the formation of nanoparticles; the band at the longer wavelength is the characteristic peak of nanoparticles. For gold metal, there was a new band at a longer wavelength between 460 and 560 nm, which is a plasmonic peak, a characteristic of AuNPs, as shown in Figure 1b [31,32]

The maximum wavelength absorption (lambda maximum (λ_max_)) of the different sizes of metal nanoparticles obtained using different reducing agents were measured. The lambda maximum (λ_max_) of the plasmonic peak varied with the reduction, as shown in Table 1. The λ_max_ of AuNPs reduced with sodium borohydride (NaBH_4_) was shorter than the λ_max_ of AuNPs synthesized using other reducing agents. This may be due to the λ_max_ dependence on the size of the nanoparticles in solution, since NaBH_4_ nanoparticles were the smallest in size.

Characterization of engineered nanoparticles with high-resolution transmission electron microscopy (HRTEM). The synthesized AuNPs were characterized with HRTEM to establish their sizes and shapes. To avoid the precipitation and agglomeration of nanoparticles, which can affect their morphology in such a way that the determination of the effect of each reducing agent on size and shape would be hampered, for HRTEM characterization fresh nanoparticles were synthesized and sonicated before analysis.

**Figure 2:**
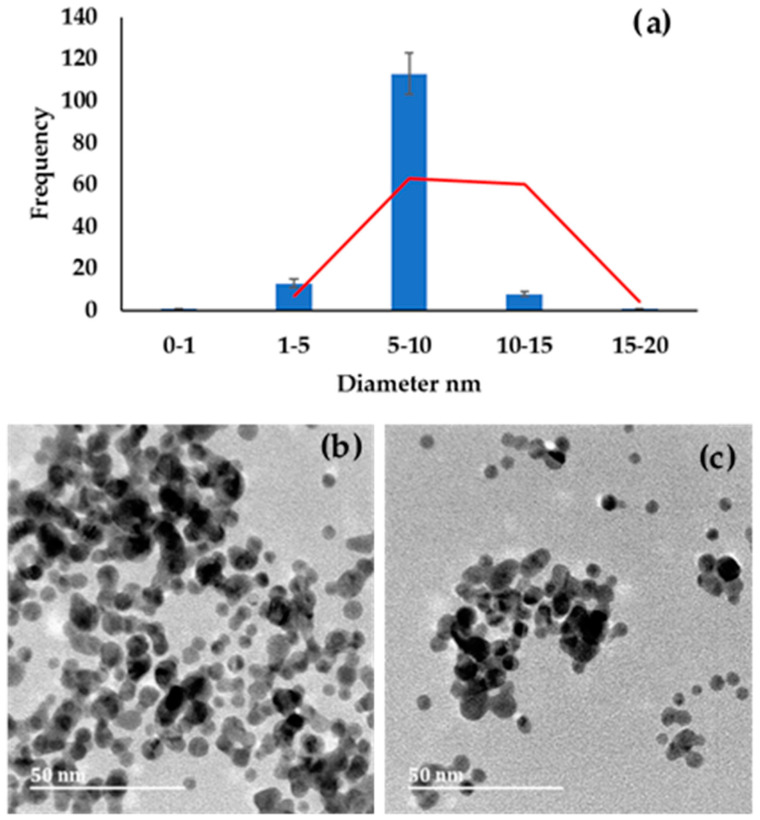
(**a**) Histogram obtained by measuring the diameter of AuNPs; (**b**,**c**) HRTEM images of sodium borohydride AuNPs.

AuNPs synthesized using NaBH_4_ as a reducing agent produced nanoparticles with an average size diameter of less than 10 nm (<10 nm). Selected HRTEM images and a plot of particle size diameter distribution are shown in Figure 2. The results obtained are comparable to those obtained by Milam et al. [32], where Au salt was reduced by NaBH_4_ and produced nanoparticles with an average size of 10 nm. Most NaBH_4_-synthesized AuNPs were spherical, and some were triangular, and their diameter ranged between 5 and 10 nm, with the average less than 10 nm.

**Figure 3:**
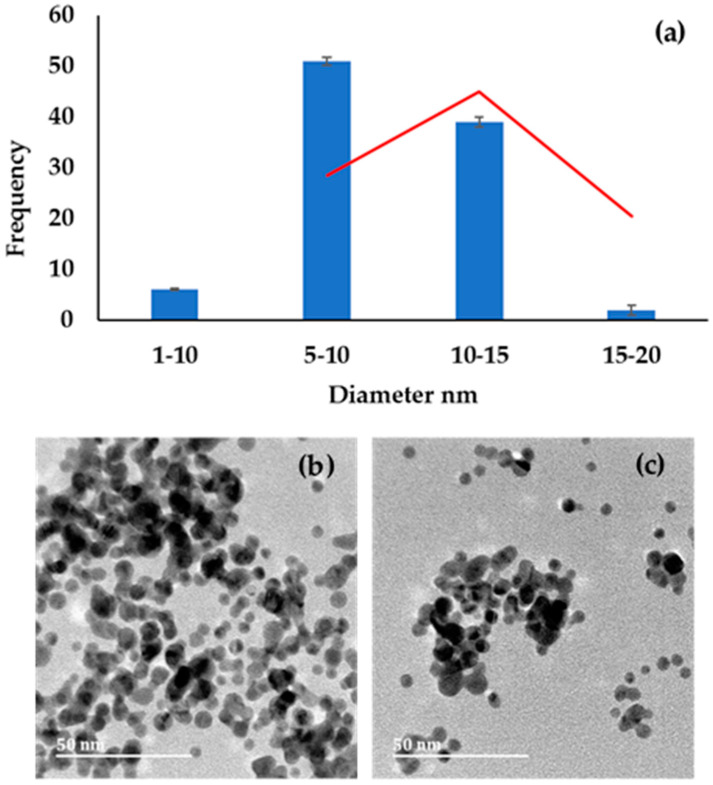
(**a**) Histogram obtained by measuring the diameter of AuNPs; (**b**,**c**) HRTEM images of ascorbic acid AuNPs.

The use of ascorbic acid in the synthesis AuNPs resulted in the generation of nanoparticles (ASCB-AuNPs) with a diameter between 5 and 15 nm. The HRTEM images and the plot of the particle size distribution of the NPs’ diameters are presented in Figure 3. The results show that the synthesized ASCB-AuNPs are spherical, but clustered due to uncontrollable agglomeration, even in the presence of tri-sodium citrate as the capping agent to induce stability. Their diameter range was between 1 and 20 nm, with an average size of 12 nm, as shown in Figure 3a. These results are comparable to those reported in the literature [32].

**Figure 4:**
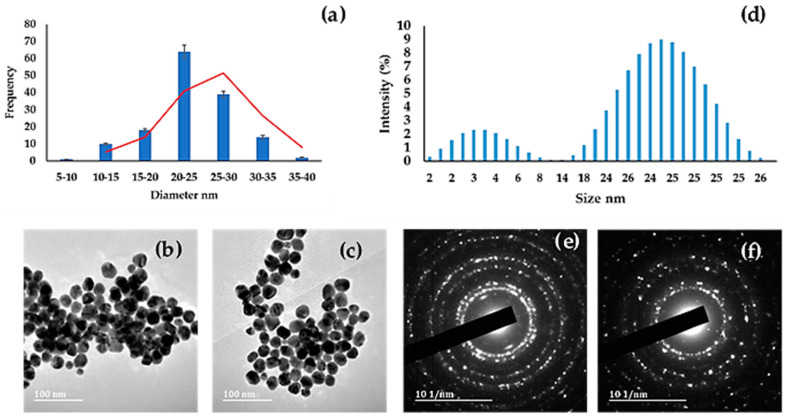
(**a**) Histogram obtained by measuring the diameter of AuNPs; (**b**,**c**) HRTEM images of tri-sodium citrate AuNPs with the (**d**) zetasizer and (**e**,**f**) selected area of electron diffraction (SAED).

The synthesized TSC-AuNPs have spherical and irregular shapes, with an average size of 27 nm (Figure 4). AuNPs synthesized with the reducing agent tri-sodium citrate (TSC-AuNPs) resulted in nanoparticles with a particle size dimension of above 20 nm [31,32]. The TSC-AuNPs were analyzed using HRTEM, and the results are presented in Figure 4b,c.

**Figure 5:**
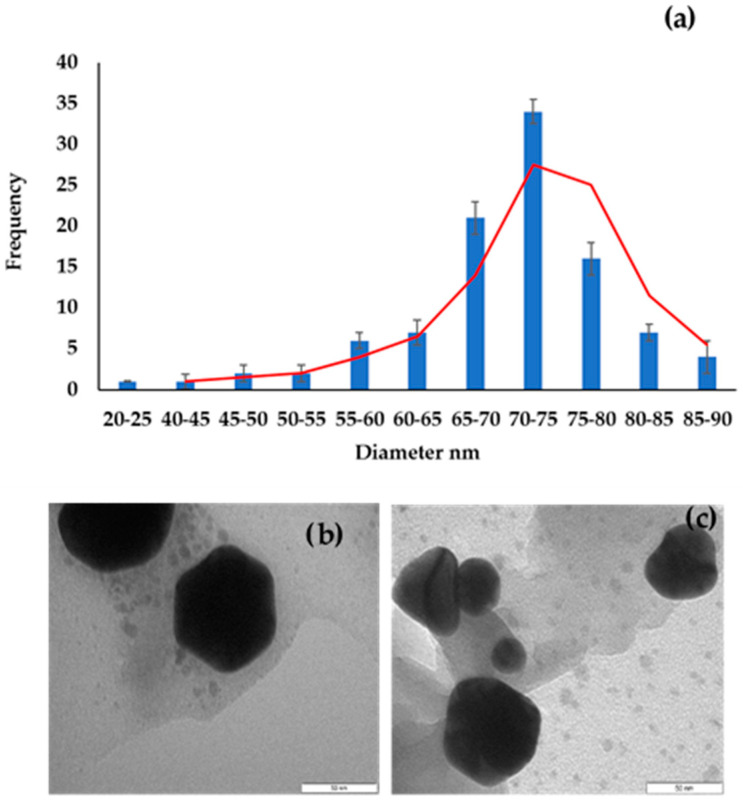
(**a**) Histogram obtained by measuring the diameter of AuNPs; (**b**,**c**) HRTEM images of seed growth-synthesized AuNPs.

Sodium borohydride (NaBH_4_) and ascorbic acid-reduced AuNPs were combined and left at room temperature they grew to a diameter of more than 20 nm. This resulting solution of NPs was sonicated for longer than other synthesized AuNPs, because of the observed agglomeration. The shapes of the AuNPs were found to have an average size of 75 nm, with spherical, triangular, and rhombic shapes, while some were irregular (Figure 5). This could be because controlling shape of nanoparticles synthesized by the seed growth method is generally difficult. AuNPs with a particle size greater than 50 nm (NaBH_4_-ASCB-AuNPs) were synthesized using the seed growth method [33,34].

### 2.2. Analysis of Gold Salts and Nanoparticles

**Figure 6:**
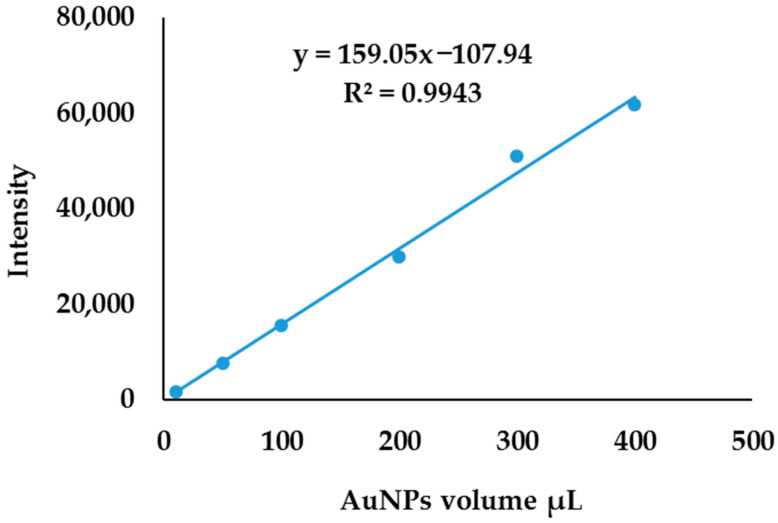
Calibration curve obtained by plotting intensity against AuNPs’ volume spiked.

The volume of AuNPs was varied to determine if the total concentration of gold is proportional to the number of nanoparticles in the solution. The stock solution made from citrate-synthesized AuNPs was stable compared to stock solutions from other reducing agents. Different standards with varying AuNP concentrations were prepared by varying the volume of AuNPs obtained from a stock solution of citrate-synthesized AuNPs. The volume was varied between 10 µL and 400 µL of AuNPs; standards were digested and diluted to 10 mL with Millipore water. The solutions were analyzed for the total concentration of gold with ICP-OES and the obtained intensities were plotted against the volume of AuNPs, as shown in Figure 6. The plot of volume versus intensity gave a linear relationship, with linear regression (R^2^) of 0.9943, this shows that the total concentration of Au in the solution is proportional to the number of AuNPs in the solution. However, this calibration curve does not reveal the total gold concentration nor the number of AuNPs in the solution. If the total gold concentration is known in the solution, a mathematical model can be developed to determine the number of nanoparticles in the solution, similar to the SP-ICP-MS algorithm method [15,16,17,35,36,37,38,39,40].

An external calibration curve based on gold salt standards was used to determine the total concentration of gold in standards prepared from varying the volume of AuNPs. The external calibration curve was obtained by weighing AuCl_4_^−^ and performing a serial dilution of stock solutions. Gold salt standards ranged from 0 mg L^−1^ to 50 mg L^−1^ and the calibration curve was linear, with linear regression of 0.993. 

**Figure 7:**
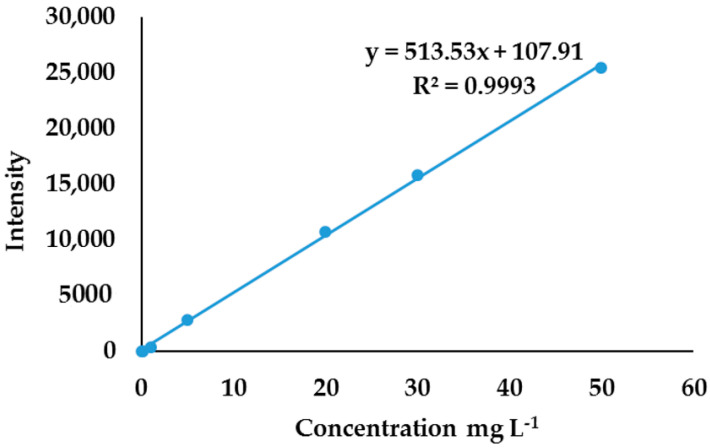
External calibration curve obtained by plotting intensity against the concentration of gold salt standard.

**Figure 8:**
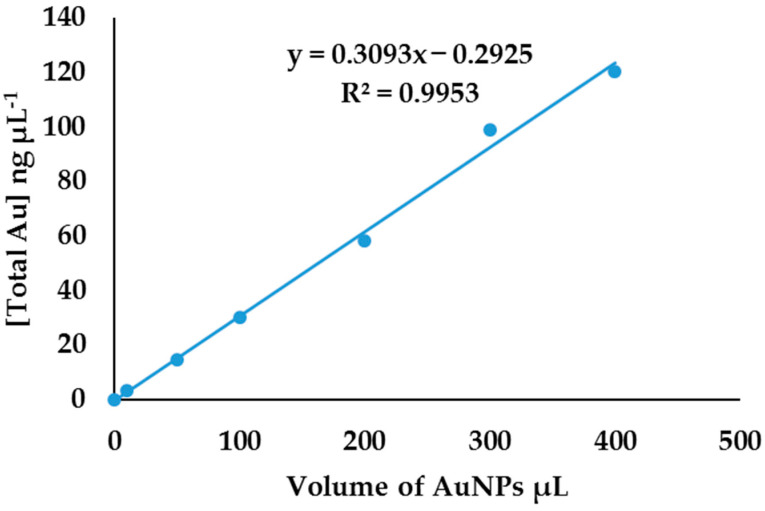
Relationship between the total concentration of AuNPs and AuNPs’ volume spiked in soil.

The total concentration of gold in AuNP standard solutions was obtained by correlating the intensities from AuNP standards (Figure 6) to the external calibration curve in Figure 7. To find the relationship between the total concentration of gold and the AuNPs’ volume, the total concentration obtained was plotted against the volume of AuNPs, as shown in Figure 8. This established relationship is linear, with a linear regression of 0.9953. This relationship confirms that the total gold concentration in a certain volume of AuNPs is directly proportional to the number of nanoparticles in the solution; when the volume of nanoparticles is changed, the number of nanoparticles changes. Knowing the shape, λ_max_, absorbance, average diameter, the volume of nanoparticles, and the total concentration, a robust analytical method for quantification of metal ENMs can be implemented that needs these parameters to be obtained experimentally, as previously suggested by other researchers. However, the problem is in the isolation of nanomaterials from bulk matrices. Unlike SP-ICP-MS which detects a single particle, ICP-OES gives the total concentration of metals; hence, a method is needed to separate nanomaterials from the matrix before analysis [21,41].

### 2.3. Extraction and Separation Methods of AuNPs

To extract nanomaterials from bulk matrices, leachate prepared as described in the experimental section was used, which mimics the relevant environmental processes to extract spiked (sediments) into supernatants. The centrifuge and phase transfer methods were then evaluated in order to determine the total concentration of gold that produces certain nanoparticles in the solution.

**Figure 9:**
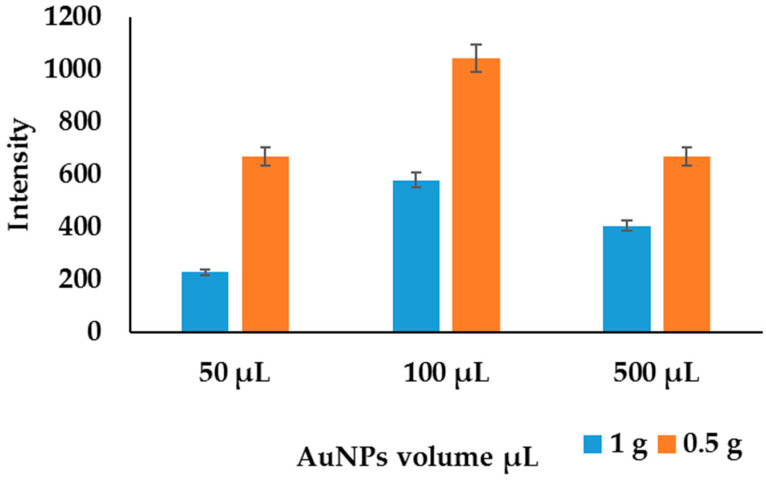
Effects of AuNPs’ volume spiked in different masses of soil.

Centrifuge method—A centrifuge separation method proposed by EI Hadri et al. [42] was used to evaluate the separation of AuNPs from supernatants. In this method, about 0.5 g and 1 g of sediments were spiked with varying volumes of AuNPs in triplicates, then extracted and separated as discussed above, followed by digestion and analysis by ICP-OES. AuNP residues obtained from the centrifuge were digested and analyzed with ICP-OES. To determine the sample mass and AuNP volume, the intensities obtained in ICP-OES were plotted against the volume of AuNPs used to spike different sample masses, as shown in Figure 9. The sample mass of 0.5 g spiked with 100 µL AuNPs showed high intensities compared to the others; 0.5 g was selected as an aliquot to be used in these analyses due to the highest concentration obtained. When 500 µL of AuNPs was spiked, there was a decrease in the intensities, and this was attributed to cluster formation, which settled with sediments during the centrifugation due to high concentration of AuNPs. Hence, volumes of AuNPs above 400 µL were removed from the calibration curve.

**Figure 10:**
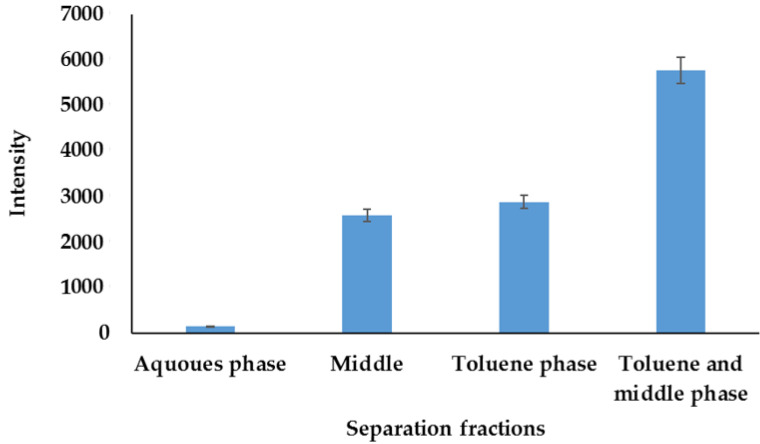
AuNP partitioning in organic, middle, and aqueous phase.

Phase transfer method—The phase transfer separation method has been demonstrated to separate nanomaterials from bulk matrices, without changing their shapes and sizes. Hence, the method was evaluated to determine its efficiency in the separation, i.e., removal, of AuNPs. The problem with the phase transfer is the location of nanoparticles in the middle phases [43,44,45]. To evaluate the phase transfer method, 20 mL of diluted supernatant from 0.5 g of sediment was spiked with 100 µL of AuNPs and was separated into 6 mL of toluene. The fraction that contained AuNPs after separation was determined by analyzing aqueous, middle, and organic phases using ICP-OES, and the results are shown in Figure 10. As previously reported, AuNPs were abundantly found in toluene and in the middle phase compared to the aqueous phase. The toluene and the middle phase residues were, therefore, combined and analyzed with ICP-OES. The traces of gold detected in the aqueous phase were attributed to the unreacted gold during the reduction. Since the gold salt is not 100% converted to AuNPs, the phase transfer separation method is useful in accurately and efficiently separating AuNPs from dissolved gold metal ions. 

### 2.4. Modeling of Nanoparticles

To develop the model to estimate the number of nanoparticles from a specific concentration of gold, a gravimetric method proposed by Shang et al. [13] was adopted in this study. Their method suggested that the number of nanoparticles in a colloidal suspension can be determined by measuring the quantities of suspensions and the unit quantity of one nanoparticle, and the molar concentration of gold nanoparticles can be subsequently calculated. The challenge, however, is how to determine the number of atoms per particle of gold in the environmental relevant concentration levels within a sample. In the present study, the parameters used by Shang et al. [13], such as the size and concentration, were not assumed as usual happens in the literature. HRTEM was used to determine the morphology of AuNPs and the average diameter. These parameters, such as the size, shape, and molar mass, were fitted in Equation (1) to find the average number of atoms per particle, which depend on the volume of the sphere. This was because the synthesized AuNPs were spherical, with an average diameter of 27 nm, which agrees with the assumption made by Liu et al. [14] that AuNPs synthesized by the citrate method are spherical. The average number of atoms per AuNP was found to be 608.66 × 10^3^ per particle.

(1) $$N\;\left(particles\right)=\frac{\pi \times dExp3\times \rho \;}{6M}\times N\left(A\right)$$

where *d* is the diameter, *ρ* is the density of gold, *M* is the molar mass, and *N (A)* is the Avogadro’s number

In this present model, the molar concentration of nanoparticles was found experimentally using the external calibration curve, as shown in Figure 7 above. The number of AuNPs was obtained from the total molar concentration of AuNPs, using Avogadro’s number, the average number of atoms per AuNPs, and the volume of AuNPs in suspension, using Equation (2). The results for experimental and calculated parameters used in modelling are shown in Table 2.

(2) $$N=\frac{C\;\times N\;\left(A\right)\times V\;}{N\;\left(particles\right)}\;$$

where *C* is molar concentration, *V* the volume of AuNPs suspension, *N(A)* is Avogadro’s number, and *N (particles)* is the number of atoms per gold nanoparticles.

**Figure 11:**
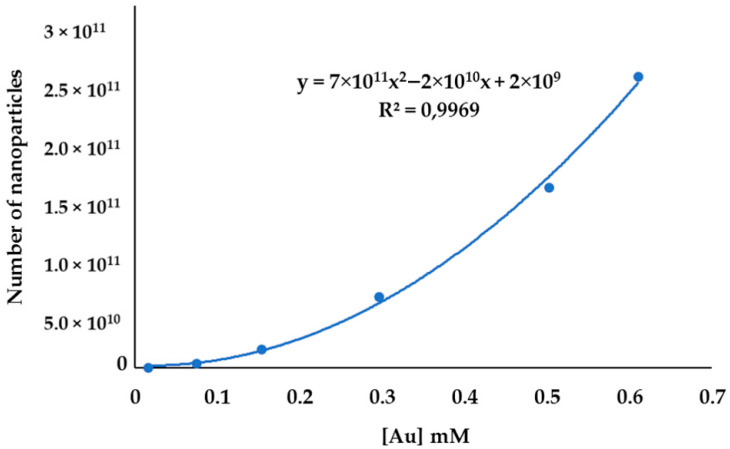
Number of AuNPs at different concentration levels.

The number of nanoparticles obtained using equation (2) was plotted against the molar concentration of gold nanoparticles; the plot was fitted into a polynomial curve, as shown in Figure 11. The plot indicates that the number of nanoparticles increases exponentially as the volume of AuNPs increases. The data were also fitted into the logarithm (number of nanoparticles), and there was no change in the curve. It can be inferred from this model that the number of nanoparticles is related to the total concentration of metal in supernatants after separation.

**Figure 12:**
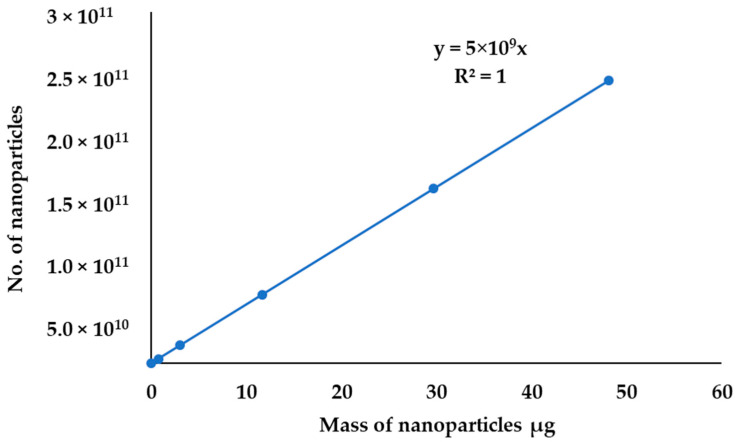
Linear relationship between the number of nanoparticles and the mass of the nanoparticles.

Since the gravimetric method is generally based on mass, a volume of AuNPs’ colloidal suspension and the molar concentration of AuNPs were used to obtain the mass of gold that makes the nanoparticle. On the plot of a number of nanoparticles against the mass of gold nanoparticles, the model gave a linear relationship (Figure 12). The linear model simplifies the estimation of the number of nanoparticles in a sample; if the mass of nanoparticles is known, the number of nanoparticles can be found. However, it is difficult to separate the measurable mass of nanoparticles in the environmental matrices using gravimetric analysis. In this study, the separation method and ICP-OES were used to find the mass of gold. This proposed method is cheaper compared to methods reported in other literature used to find the concentration and mass of nanoparticles [19,21,22,36,39,46]. 

The developed model was compared with other mathematical models reported in the literature. Most of the reported models need the diameter and the shape of nanoparticles, which is obtained from the HRTEM measurements, as shown in Table 3. Since SP-ICP-MS can measure the size and estimate the shape of nanoparticles in situ, HRTEM is not a requirement in this model [18,23,24]. Furthermore, for the model to be applied in environmental samples, it requires the separation of nanoparticles from bulk matrices. The centrifuge is the most extensively used extraction technique for nanoparticles in environmental samples. However, most researchers do not apply the model to environmental samples; as a result, an extraction step is not required. In the current model, the molar concentration of AuNPs is measured with ICP-OES and the mass of AuNPs is obtained by using the colloidal suspension volume, which is related to the number of AuNPs. ICP-OES is sensitive and widely available, and this model uses a simple method of detection and quantification of nanoparticles in a sample. 

## 3. Materials and Methods

### 3.1. Chemicals, Reagents, and Standards

Nitric acid ≥ 62%, hydrochloric acid ≥ 37%, gold (III) chloride trihydrate ≥ 99.99%, L-ascorbic acid, sodium borohydride ≥ 99%, octadecylamine, sodium bicarbonate, tri-Sodium citrate, calcium sulphate hydrate, magnesium sulphate, and potassium chloride were purchased from Sigma Aldrich (Steinheim, Germany). Toluene, methanol, and acetone were of analytical grade from Sigma Aldrich and used without further purification. 

### 3.2. Instruments

For high-resolution transmission electron microscopy (HRTEM), a JEOL JEM-2100F transmission electron microscope (JEOL, Beijing, Shanghai, China) was used to measure the size and shape of ENMs. An ocean optics spectrometer (model HR2000+ manufactured by Ocean Optics at EW Duiven, The Netherlands) equipped with spectra suite software (Ocean Optics, Duiven, The Netherlands) was used to measure the wavelength of ENMs. The light source used was a tungsten halogen product of ocean optics (Ocean Optics, Duiven, The Netherlands). The light source was connected to the detector by a fiber optic cable (cables were from Ocean Optics—600-2-vis-BX model 727-733-2447, with a range of 400–2100 nm). Inductively coupled plasma optical emission spectrometry (ICP-OES) model 5300DV was bought from Perkin Elmer, Waltham, United States of America, and it was used to measure the concentration of ENMs in the model samples.

### 3.3. Preparation of Metal Standards Solutions and Nanomaterials Stock Solutions

A 100 mg L^−1^ stock solution of Au was prepared by dissolving 0.01 g of Au salt in 100 mL of water. The working standard solutions with a concentration range of 0.01–1 mg L^−1^ were then prepared from the stock solution and used for the calibration of the ICP-OES. 

**Figure 13:**
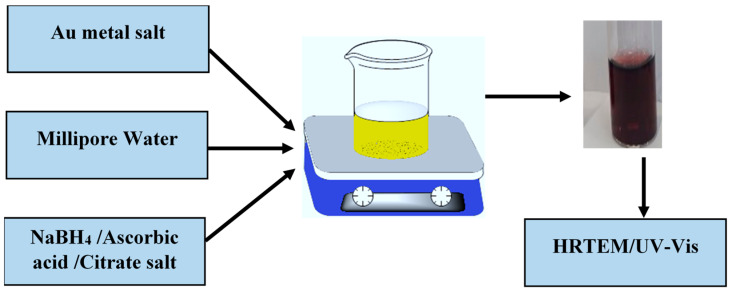
Synthesis of metal engineered nanoparticles using different reducing agents.

Engineered AuNPs were prepared at different reaction conditions. Parameters such as reducing agent, temperature, stirring conditions, and synthesis times were varied to obtain different size dimensions of nanoparticles. All nanomaterials were prepared using fresh Millipore water. The obtained AuNPs were maroon red in color, as shown in Figure 13.

Synthesis of 5–10 nm engineered nanoparticles (sodium borohydride)—0.01084 M of gold (Au) solution was prepared by dissolving 0.4267 g of HAuCl_4_.H_2_O salt in 100 mL of Millipore H_2_O. The gold solution (600 µL) was mixed with 10 mL of 1.356 mM tri-sodium citrate solution, followed by the addition of a freshly prepared 0.1864 M NaBH_4_ (20 µL) at room temperature (25 °C) while stirring; the sodium borohydride was used as a reducing agent, while sodium citrate was used as the stabilizing agent. The solution turned purplish brown with a little touch of maroon. The resulting AuNPs were characterized using HRTEM and UV-vis.

Synthesis of 5–15 nm engineered nanoparticles (ascorbic acid)—To make a solution of AuNPs with an average size of 10–15 nm, in an Au-tri-sodium citrate solution prepare above, a freshly prepared 0.04580 M ascorbic acid (20 µL) was added as a reducing agent at room temperature (25 °C) while stirring; the solution immediately turned dark maroon red. The resulting AuNPs were characterized using HRTEM and UV-vis.

Synthesis of 20–30 nm engineered nanoparticles (citrate)—A 2.986 mM Au solution was prepared from HAuCl_4_.H_2_O salt by dissolving about 0.4699 g of the gold salt in 400 mL of Millipore H_2_O. The resulting solution was heated on the hotplate to approximately 100 °C, and, thereafter, 10 mL of 0.2740 M tri-sodium citrate was added to the boiling solution. The solution was stirred while observing the color changes. The color transition was from yellow to colorless and then to deep red. The resulting solution was immediately taken off the hotplate, cooled to room temperature (25 °C), and then transferred to a 2000 mL volumetric flask, and made up to the mark with Millipore water, followed by thorough mixing. The resulting AuNPs were characterized using HRTEM and UV-vis.

Synthesis of 65–80 nm engineered nanoparticles (seed growth method)—AuNPs with an average size of 65–80 nm were synthesized by reducing Au solution (0.01084 M) with the addition of 60 µL of sodium borohydride (NaBH_4_) with constant stirring, in the presence of AuNPs synthesized by ascorbic acid. Seed AuNPs (5–10 nm, 50 µL) were mixed with seed growth solution (5–15 nm, 2 mL) and then the solution was added dropwise to 10 mL of Au solution to allow the growth of AuNPs. The resulting solution was brown and faintly pink, and was characterized using HRTEM and UV-vis. 

### 3.4. Sampling and Sample Preparation 

Samples were collected along the Mgeni River. Sediment samples were scooped from the mainstream of the river into clean aluminum foil, labelled, and stored in a cooler box kept under 4 °C, and transported to the laboratory. Samples were dried and homogenized with a pestle and mortar. The samples were then sieved with a mesh size of 0.53 µm and were kept in a dark and cool place for further analysis.

### 3.5. Extraction of AuNPs in Sediments

The leachate was prepared according to the method described by El Hadri et al. [42]; as adapted from environmental protection agency (EPA) recommendations, the moderately hard water was prepared by weighing NaHCO_3_ (0.096 g = 96 mg L^−1^), CaSO_4_.H_2_O (0.06 g = 60 mg L^−1^), MgSO_4_ (0.06 g = 60 mg L^−1^), and KCl (0.04 g = 40 mg L^−1^) to make an extraction leachate [1,42]. The resulting leachate had an ionic strength of 3.3 mM. A 0.5 g of sieved sediment aliquot was weighed into a round-bottom flask and 10 mL of leachate was added to the sediments. AuNPs were spiked into sediments by addition of small volumes, ranging from 10 to 500 µL of the sediments–leachate system.

Centrifuge separation of AuNPs—The sediment-leachate systems were transferred into 50 mL centrifuge tubes and were centrifuged at 2000 rpm for 20 min to separate the supernatant from the sediments. This method assumes that the particles in suspension are spherical and behave according to Stokes’ law; therefore, since the system was spiked with spherical AuNPs, this extraction method should extract spherical nanoparticles into the supernatant phase. The supernatants were decanted into a 50 mL beaker.

Phase transfer separation of AuNPs—A 0.3354 g octadecylamine (OCTDA) was weighed and dissolved in 100 mL of toluene to make a 0.01 M solution. A 6 mL OCTDA toluene solution was added into aqueous supernatant containing extracted AuNPs and diluted to a final volume of 26 mL with Millipore water. The mixture was transferred into a 50 mL separating funnel and agitated for 20 min to allow the transferring agent (OCTDA) to tag AuNPs into the toluene phase. The mixture was left for 2 h for the two phases to separate. After separation, the toluene phase was left in a fume hood to evaporate to dryness in a 50 mL beaker. The dried residues were digested by 4 mL of aqua regia in room temperature overnight. The concentrates were transferred into ICP tubes and diluted with Millipore water to 2% aqua regia. The total Au concentration in the extract was analyzed with ICP-OES. The efficiency of the phase extraction procedure was verified by digesting the known concentration of AuNPs without extraction by OCTDA toluene solution. 

Digestion of residues and ICP-OES analysis—The AuNP supernatant residues were digested by adding 4 mL of undiluted aqua regia (1:3; HNO_3_:HCl) to the extracts in a beaker, and the solution was left overnight at room temperature in a fume hood. The digested AuNPs were transferred from the beaker into the ICP tube and diluted with deionized water to reach 2% aqua regia. The total Au concentration was determined by ICP-OES. The efficiency of the centrifuge extraction procedure was verified by digesting extracts of the spiked soil.

## 4. Conclusions

In this study, useful modeling information for environmental scientists involved in the investigation of nanoparticle occurrence, where the molar concentration of nanoparticles and the number of nanoparticles are needed in quantitative analysis, is provided. AuNPs were synthesized and characterized to be used in modeling. The method uses leachate to extract nanoparticles from bulk matrix, centrifuge, and phase transfer techniques for separation of AuNPs from the bulk matrix. HRTEM and ICP-OES were used to measure size and molar concentration, respectively. These parameters were fitted to the adapted gravimetric formulas to obtain the number of AuNPs in a sample. This has demonstrated that the mass of AuNPs is related to the number of AuNPs in a sample, and this established relationship can be used to determine the number of nanoparticles in the environment. This model simplifies the study of nanoparticles in the environment by HRTEM and ICP-OES, which are readily available techniques in many laboratories.

## Figures and Tables

**Table 1:** Synthesized AuNPs using different reducing agents.

	AuNPs			
Reducing Agent	Ascorbic Acid	Tri-Sodium Citrate	Sodium Borohydride	Seed Growth
Average diameter nm	5–15	20–30	5–10	65–80
λ_max_	524.94	532.38	520.95	562.03

**Table 2:** Synthesized AuNPs by different reducing agents.

Volume µL	Concentration mM	Mass µg	No. of NPs
10	0.016624	0.032750	164,478,413.8
50	0.074379	0.73263	3,679,510,010
100	0.15330	3.0200	15,167,634,649
200	0.29613	11.668	58,598,379,921
300	0.50264	29.706	1.49193 × 10^11^
400	0.61058	48.113	2.4164 × 10^11^

**Table 3:** Different methods from the literature to determine the concentration of nanoparticles.

Instrument	Model	Nanoparticle	Parameter	Extraction Method	Reference
ICP-MS	Analysis of different fractions	Ti, Ag	Concentration	Centrifuge, evaporation, ultrasonic	Polesel et al. [45]
Sp-ICP-MS ICP-MS	N_NP_ = [f_NP/_9Q_sam_ × η_n_)]	Ag	Concentration	Centrifuge	Aznar et al. [23]
	N_NP_ = [f_NP/_9Q_sam_ × η_n_)]	Au, Ag	Size distribution, particle number concentration, diameter	Centrifuge, ultrasonication	Yang et al. [17]
	N_NP_ = [f_NP/_9Q_sam_ × η_n_)]	Ag	Nanoparticle size,	Centrifuge	Pace et al. [46]
Microgravimetry TEM	C_m_ = ∆_m_S/υ	Si	Concentration, extinction coefficient,		Reipa et al. [14]
UV-vis TEM	A = πR^2^Q_ext_d_0_N/2.303	Au	Size, concentration, absorbance		Haiss et al. [36]
UV-vis TEM	C = A/ԑd_0_	Ag	Concentration, extinction coefficient, size, absorbance		Paramelle et al. [15]
DLS TEM	N(d) = n_b_ – C_1_ + C_2_/d_n_	Ag, Si	Number density, extinction		Levin et al. [16]
UV-vis TEM	C = (α/N)	Nanosphere Polystyrene	Diameter, volume, concentration		Niskanen et al. [39]
ICP-OES, HRTEM, UV-vis	N = (CxN_a_ × V)/N_particles_	Au	Concentration, number of AuNPs, mass of AuNPs, diameter	Leachate, centrifuge, phase transfer	Current method

## Data Availability

All data will be made available upon request.

## Sample Availability

Environmental sample residues are available from authors.
